# Complete Genome Sequence of a Porcine Epidemic Diarrhea S Gene Indel Strain Isolated in France in December 2014

**DOI:** 10.1128/genomeA.00535-15

**Published:** 2015-06-04

**Authors:** Béatrice Grasland, Lionel Bigault, Cécilia Bernard, Hélène Quenault, Olivier Toulouse, Christelle Fablet, Nicolas Rose, Fabrice Touzain, Yannick Blanchard

**Affiliations:** a Anses, Laboratory of Ploufragan/Plouzané, Unit of Viral Genetics and Biosafety, Ploufragan, France; b Clinique VET Flandres, Hazebrouck, France; c Anses, Laboratory of Ploufragan/Plouzané, Unit of Porcine Epidemiology and Welfare, Ploufragan, France

## Abstract

We report the first and only case of a porcine epidemic diarrhea (PED) outbreak occurring in December 2014 in northern France, and we show using the full-length genome sequence of the French PED virus (PEDV) isolate that it was a PEDV indel strain close to German PEDV strains recently isolated.

## GENOME ANNOUNCEMENT

Porcine epidemic diarrhea (PED) was first described in England in 1971 and then throughout Europe until the end of the 1990s. The etiologic agent is an alphacoronavirus, the PED virus (PEDV) (1). In France, no PED cases have been described since the late 1990s, and the immune status of the pig population against PEDV is low (2). Since April 2013, a severe epizooty of PED, characterized by watery diarrhea and vomiting, has been striking the United States. Suckling piglets are the most affected, with 90 to 95% mortality rates. In France, as a first measure for the control of a potential outbreak, PED has been classified in the list of the first category of animal health hazards, making notification to veterinarian officers compulsory (ministerial order of 12 May 2014 of the French Ministry of Agriculture that modified Annex Ib of the Ministerial order of 29 July 2013). Here, we report the sequence of the full-length genome of an indel strain isolated in the first PED case in northern France in December 2014.

The outbreak occurred in a farrow-to-finish herd. The mortality rate ranged between 3.3 and 5.5% in the fattening building and reached 12% in piglets after 1 week and 25% at weaning in the farrowing building. The samples collected were from the jejunum of 3 affected animals that had died within the day. Next-generation sequencing and data analysis were performed on RNA extracted from the 3 jejunum samples in order to obtain the full-length genome sequence. Trimmomatic and TMAP were used for cleaning and aligning reads (3) and SAMtools to create a consensus sequence (4). Alignment was realized using progressiveMauve (5). The PEDV genome sequences obtained from jejunum samples were named PEDV FR/001/2014 to FR/003/2014. No single-nucleotide variants were observed between the sequences obtained from the three jejunum samples. PEDV FR/001/2014 was found to be genetically related to the recent German strain GER/L00719/2014 (99.9% identity), isolated in May 2014 (6), and with USA/Indiana12.83/2013 (99.8% identity), a U.S. strain detected in June 2013 (7). The USA/OH851/2014 strain shared 99.4% identity with the German and French isolates (6). Two major types of PEDV strains are circulating in the United States and Asia. The first PEDV strains isolated in the United States in April 2013 present 99.5% nucleotide identity with a Chinese strain, China/AH2012, isolated in 2012 (8, 9), and are qualified as highly pathogenic strains. The second strains were identified later and present both an insertion and a deletion in the S gene and thus are called indel strains (7, 10). The first U.S. indel strain, named OH851, was described in January 2014 (10), and another PEDV indel variant named USA/Indiana 12.83/2013 was described in June 2013, suggesting that highly pathogenic strains and indel strains were introduced at the same time in the United States (7). According to our results, it appears that the French and German PEDV isolates are more related to the USA/Indiana 12.83/2013 strain than to the USA/OH851/2014 isolate. This might suggest that indel strains circulate in Europe for a longer time than was previously hypothesized, a time that might even be contemporary to the introduction of PEDV to the United States around mid-2013.

### Nucleotide sequence accession number. 

The PEDV FR/001/2014 genome sequence has been deposited in GenBank under the accession no. KR011756.
